# Toward the Ultimate-High-Speed Image Sensor: From 10 ns to 50 ps

**DOI:** 10.3390/s18082407

**Published:** 2018-07-24

**Authors:** Anh Quang Nguyen, Vu Truong Son Dao, Kazuhiro Shimonomura, Kohsei Takehara, Takeharu Goji Etoh

**Affiliations:** 1 School of Electronics and Telecommunications, Hanoi University of Science and Technology, 1 Dai Co Viet Road, Hanoi 100000, Vietnam; quang.nguyenanh@hust.edu.vn; 2 Department of Industrial and Systems Engineering, International University, Vietnam National University HCMC, Quarter 6, Linh Trung Ward, Thu Duc District, Ho Chi Minh City 720400, Vietnam; dvtson@hcmiu.edu.vn; 3 School of Science and Engineering, Ritsumeikan University, 1-1-1 Noji-Higashi, Kusatsu, Shiga 525-8577, Japan; skazu@fc.ritsumei.ac.jp; 4 School of Science and Engineering, Kindai University, 3-4-1 Kowakae, Higashiosaka City, Osaka 577-8502, Japan; takehara@civileng.kindai.ac.jp

**Keywords:** image sensor, high-speed, multi-collection-gate, backside-illuminated, BSI, MCG

## Abstract

The paper summarizes the evolution of the Backside-Illuminated Multi-Collection-Gate (BSI MCG) image sensors from the proposed fundamental structure to the development of a practical ultimate-high-speed silicon image sensor. A test chip of the BSI MCG image sensor achieves the temporal resolution of 10 ns. The authors have derived the expression of the temporal resolution limit of photoelectron conversion layers. For silicon image sensors, the limit is 11.1 ps. By considering the theoretical derivation, a high-speed image sensor designed can achieve the frame rate close to the theoretical limit. However, some of the conditions conflict with performance indices other than the frame rate, such as sensitivity and crosstalk. After adjusting these trade-offs, a simple pixel model of the image sensor is designed and evaluated by simulations. The results reveal that the sensor can achieve a temporal resolution of 50 ps with the existing technology.

## 1. Introduction

The fundamental performance of image sensors is represented by the intensity resolution (sensitivity), the spatial resolution and the temporal resolution, and their dynamic ranges. For scientific applications, the spectrum resolution is also important. The resolution limits are governed by noise. The lower limit of the sensitivity is the noise level on the signal intensity. The temporal and the spatial resolutions are limited by temporal and spatial mixing of signals. However, the infinitesimal resolution cannot be achieved even without noise.

The lowest value of the sensitivity is one electron generated by one photon, if the quantum efficiency (the number of generated electrons divided by the number of incident photons) is close to one. The spatial resolution limit of microscopes with green light is about 0.3 µm, as calculated by the Rayleigh criterion for the spatial resolution. The authors theoretically derived the expression of the temporal resolution limit of photoelectron-conversion layers. The limit of the temporal resolution of greet light is 11.1 ps for silicon image sensors [1,2]. The spectrum resolution limit may be defined from the quantum theory. However, the authors have not found a good criterion to express the limit.

In Table 1, the theoretical limits of the intensity, spatial and temporal resolutions are compared with the state-of-the-art resolution limits. Currently, the advanced image sensors have achieved the lowest readout noise level of about 0.3 e^−^ rms [3,4], and the minimum pixel size of about 1 µm [5,6,7]. The sensitivity and the spatial resolution are close to the theoretical limits. As the fastest multi-framing silicon image sensor in the world, the sensor presented in this paper operates at a frame interval of 10 ns, of which the temporal resolution is enhanced by three orders of magnitude compared with that of silicon image sensors. Nevertheless, the temporal resolution limit still remains challenging.

The dynamic range, defined as the ratio of the maximum measurable range to the resolution limit, is another performance index, which has no theoretical upper limit. The dynamic range of the signal intensity, i.e., the SN ratio, should be as high as possible. More pixel count and more frame count are preferred.

The authors proposed the Backside-Illuminated Multi-Collection-Gate image sensor (BSI MCG image sensor) [10]. The test sensor captured five consecutive frames with 600 kpixels at a frame interval of 10 ns. They also developed a special driver circuit named the ROXNOR circuit, consisting of a ring oscillator and the XNOR circuits [11]. The driver chip with the ROXNOR circuits attached to the BSI MCG image sensor chip drives the pixel groups on the sensor chip at 1 ns.

In the process of designing the sensor, the causes for degrading the frame rate were identified, which led us to derive the expression of the theoretical resolution limit of photoelectron-conversion layers. For the silicon layer illuminated with the incident green light wavelength of 550 nm at 300 K, the temporal resolution limit is 11.1 ps. The corresponding frame rate is 90.9 Gfps [1,2].

In the process of deriving the expression, the conditions for the relevant elements to minimize the total temporal resolution have been specified. A high-speed image sensor designed with consideration of the theoretical derivation could achieve the frame rate close to the theoretical limit. However, some of the conditions conflict with performance indices other than the frame rate, such as sensitivity and crosstalk. After adjusting these trade-offs, a simple pixel model of the image sensor was designed and the performance was evaluated by simulations. The results revealed that the sensor could achieve a temporal resolution of 50 ps with the existing technology. Generally, theoretical analyses not only satisfy scientists’ interests, but also play an important role in the innovation of practical products by providing insights into the underlying physics.

The paper reviewed the evolution from the invention of the BSI MCG image sensors to the proposal of a multi-framing image sensor operating at a temporal resolution of 50 ps.

## 2. Backside-Illuminated Multi-Collection-Gate Image Sensor

### 2.1. Structure and Operation

**Figure 1:**
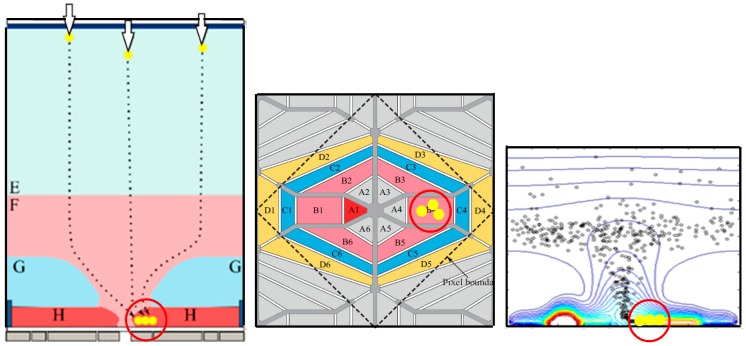
A pixel of the BSI MCG image sensor. **Left**: cross section; **center**: plane structure (A: collection/drain gates, B: storage gates, C: barrier gates, D: transfer gates; dashed line: optical 1 pixel); **right**: an example distribution of electrons at a certain time after an instantaneous illumination.

The BSI MCG image sensors incorporate two distinctive structures into each pixel [5]:

(1) The special backside illumination structure with the p-well (G in Figure 1) provides additional advanced functions on the front side under the p-well, as well as the high sensitivity with a 100% fill factor.
(2) The multiple collection gates like flower-like petals at the center of the front side (A1–A6 in Figure 1), enable imaging at very short frame intervals, specified by the temporal spread of the arrival time of signal electrons travelling from the backside layer to one of the collection gates.

The gate collecting the electrons is referred to as “a collecting gate” (A2–A6). There are six collection gates; among them, five are used for the collection gates (A2–A6) and one is employed for the drain gate (A1). The VH is applied to the five collection gates in turn at short-time intervals. Each of the storage gates (B2–B6) is attached to each collection gate. Before and after an image capture operation, the VH is applied to the drain gate (A1) to drain incoming charges, such as the dark current, to the drain (B1) and to the outside of the chip. Each pixel is surrounded by the CCD transfer gates (D1–D6). The barrier gates (C1–C6) separate the storage gates and the drain from the transfer gates. The drain also allows the electronic shutter operation for the frame interval of equal to or less than 20 ns.

**Figure 2:**
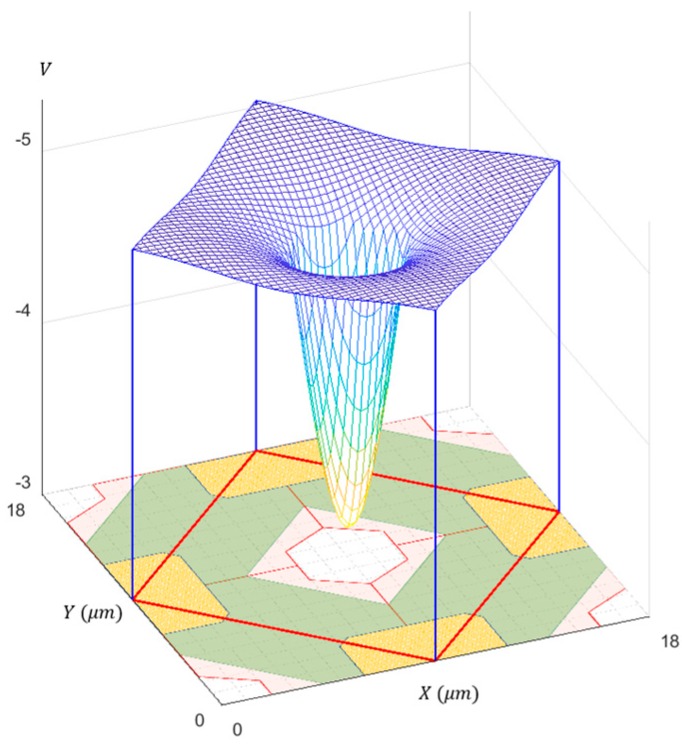
The highest potential over the p-well (5 µm > z > 3 µm). Approximate linearization of the horizontal potential profile over the p-well toward the center hole is designed by implanting Boron irons with three different masks and different implanting energies. The masks are shown at the bottom: p-well 1: a red square with a hexagonal hole; p-well 2: a moss green square with a square hole; and p-well 3: 4 brown triangles.

The p-well allows the potential separation of the upper photoelectron conversion layer and the lower circuit layer. The p-well is created by implanting Boron ions three times with different masks and different implantation energies to realize approximate linearization of the horizontal potential profile over the p-well toward the center hole, as shown in Figure 2.

The lower field causes the larger diffusion during the travel of the electrons. Therefore, the minimum field along the travel route should be maximized to minimize the total diffusion of the travel time, which requires a constant field, i.e., the linear potential profile. A similar statement was first made by Etoh et al. [12] and also applied in Dao et al. [13].

Signal electrons generated by incident photons travel toward the center hole of the p-well along the built-in horizontal electric field over the p-well via the photoelectron-conversion layer, and move downward through the hole, and are collected by the collecting gate. The collected signal electrons are immediately transferred to the storage gate attached to the collecting gate to form a signal packet. After five consecutive image packets are captured, they are read out from the pixel through the transfer gates and are transferred to a buffer memory outside the sensor at a relatively low readout rate.

### 2.2. Test Sensor

The specification of the test sensor is tabulated in Table 2. The shortest frame interval is 10 ns (equivalent to 100 Mfps). The driving voltages to drive the collection gates are delivered from the sides of the chip through the package, the socket and the circuit board, seriously elongating the RC delay.

At the next stage, the driver chip is stacked to the front side of the prototype sensor chip [6]. After the stacking, the frame interval is reduced to one nanosecond. The pixels are staggered with a pitch of 18 µm, in which the even-column pixels are shifted by a half-pitch relative to the odd-column pixels. The pixel shape is a flattened hexagon, but optically a rotated square with each side of about 12.73 µm, as depicted with the dashed lines in Figure 1, and the red lines in Figure 2. The pixel count (589,824) is about 600 kpixels. The interlace operation doubles the frame count to 10 frames, sacrificing the pixel count to a half, about 300 kpixels. The signal packets captured in repetitive imaging can accumulate in the storage gates.

**Figure 3:**
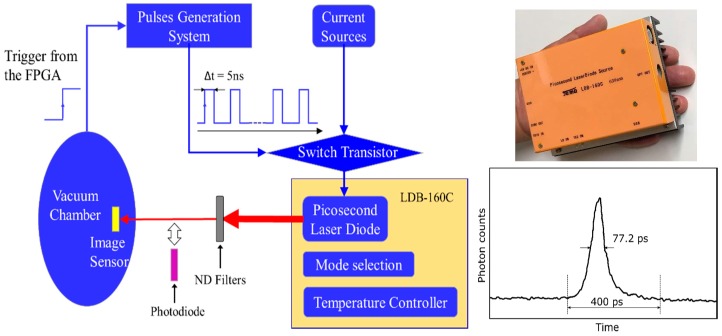
The imaging system and the LD (λ = 639 nm) used in evaluation of the temporal resolution.

A laser diode (LD) is applied to evaluate the temporal resolution. The whole duration of the laser pulse was 400 ps (<<10 ns), as shown in Figure 3. A handmade 6 × 6 LED array was also used to observe image quality, avoiding speckle noise associated with the laser illumination.

**Figure 4:**
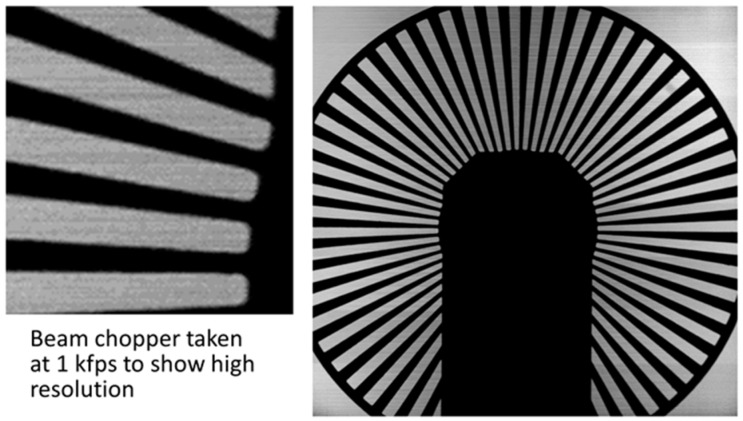
Evaluation of the spatial resolution: a laser beam chopper taken at 1 kfps. High resolution is achieved with 600 kpixels as an ultra-high-speed image sensor.

Figure 4 shows an image of a rotating laser chopper taken with the test camera and the LED array at 1 kfps, which provided the high spatial resolution with the pixel count of about 600 kpixels.

**Figure 5:**
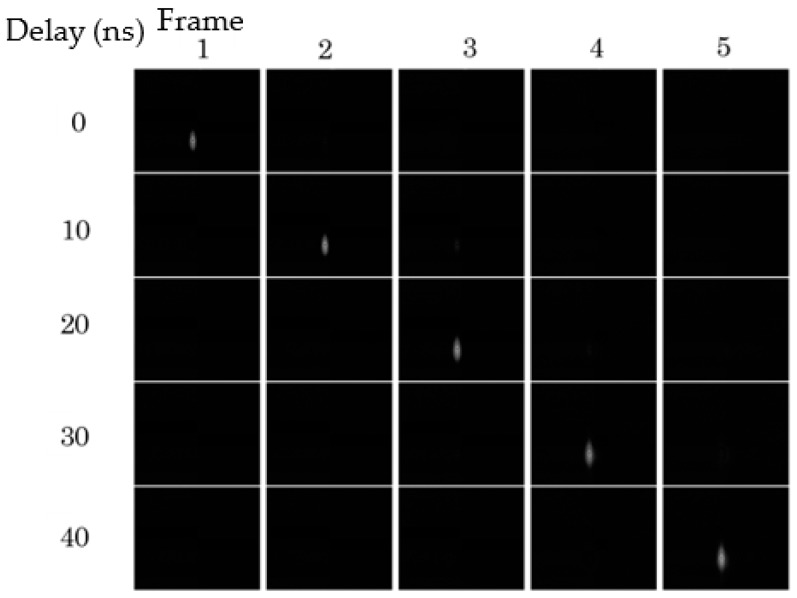
Evaluation of the temporal resolution: LD is applied in the middle of each frame (see Figure 6). Each row shows a sequence of five consecutive images; one LD image appears in each sequence.

**Figure 6:**
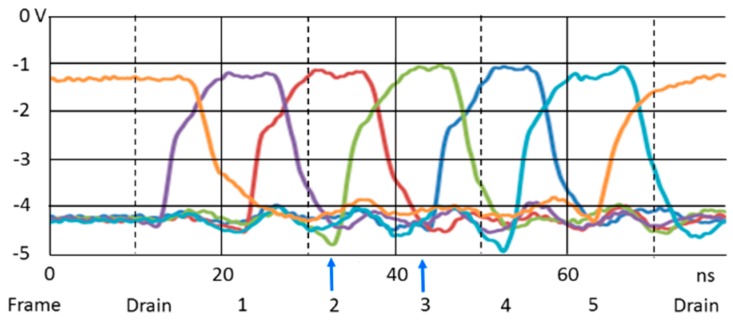
Driving pulses for the drain gate and the collection gates 1 to 5 (∆t = 10 ns). The blue arrows show the times of the LD images in the second and the third rows in Figure 5, and those in the first and the sixth rows in Figure 7.

Figure 5 shows five image sequences taken at ∆t = 10 ns with a one-pulse LD shot applied to one of the five consecutive frames at the center of each frame interval. For example, when the second and the third sequences were captured, the LD was applied at the same time, shown by the blue arrows in Figure 6. The LD image only appears in one frame for each sequence, which proves the temporal resolution of 10 ns. At the highest frame interval, temporal crosstalk appeares in the consecutive images due to the overlap of the driving voltage pulses, as shown in Figure 6.

**Figure 7:**
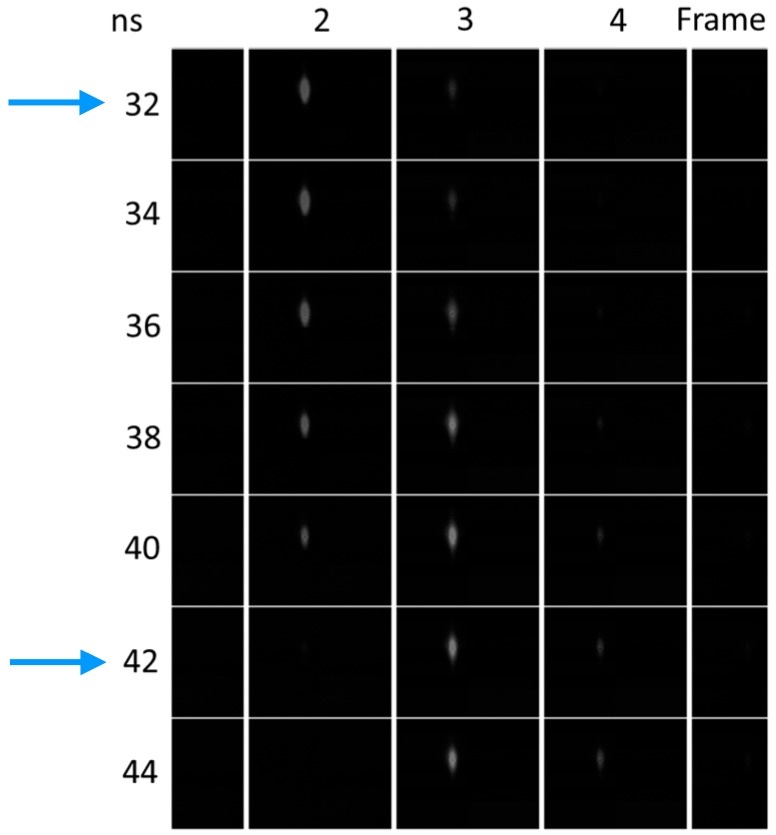
Ghost images appearing for the shifted pulses by every 2 ns. The ghost images for ∆t = 10 ns is mainly caused by the RC delay of the driving voltages, shown in Figure 6. If a short-pulse laser illumination is applied at ∆t = 10 ns or the electronic shutter is applied at ∆t > 20 ns, they may almost disappear.

Figure 7 shows the ghost images appeared in the consecutive frames due to the crosstalk, when the laser pulses were applied with the shift of every 2 ns at the shortest frame interval of 10 ns. When the frame interval increased, the overlap of the driving voltages decreased and the temporal crosstalk became smaller. At the frame interval longer than 20 ns, the drain operation between the frames for the electronic shutter was applied and the ghosts almost disappeared. The crosstalk can be mitigated by post data processing [14,15].

## 3. Design with Theoretical Consideration

### 3.1. Theoretical Highest Frame Rate

The temporal resolution can be analyzed based on the current dynamics described by the Schockley–Ramo’s theorem [16]. However, it is difficult to quantitatively detect the very weak current generated by motions of signal electrons and holes. Therefore, a common image sensor stores signal electrons in a storage element for a certain duration and measures the number of the stored electrons as a voltage. In this case, the temporal resolution depends on the spread of the arrival-time distribution of the electrons to the storage element. The effect of the motion of the holes can be neglected for the first-order approximation if the density of the electron-hole pairs is not large. The authors derived the expression of the temporal resolution limit of silicon image sensors under the condition of Reference [1].

The temporal resolution is limited by the distribution of the arrival time of signal electrons from the backside area to the front side. There are two factors to distribute the arrival time of the electrons: mixing of electrons and pure diffusion due to the random motion. For example, an electron generated near the pixel boundary travels a longer horizontal distance before arriving at the center hole than that generated at the center of the hole. Mixing of these electrons results in a large spread of the distribution of the electron arrival time.

**Figure 8:**
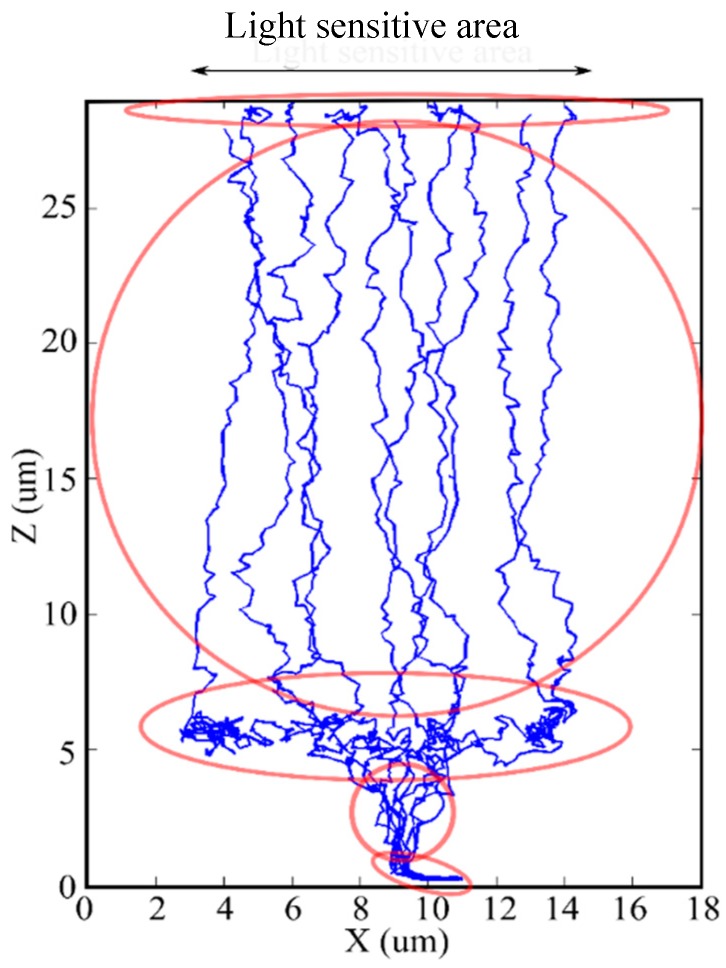
An example of trajectories of signal electrons for a pixel shown in Figure 1, which are simulated by Monte Carlo simulation.

Figure 8 shows the trajectories of signal electrons simulated by Monte Carlo simulation. The electrons are generated in the backside layer, travel vertically, and move horizontally to the center along the upper periphery of the p-well. The horizontal field over the p-well is much weaker than the vertical field, causing a large spread of the arrival time distribution. Therefore, reducing the horizontal motion is an effective way to reduce the temporal resolution. The effects of the horizontal motion can be suppressed by design efforts, as shown in the next section. They are neglected in the following theoretical analysis.

On the other hand, the effects of the vertical motion on the distribution of the arrival time cannot be eliminated. The effects are twofold: the distribution of the penetration depth of light and the vertical random motion of electrons. The penetration depth distribution causes the distribution of the average vertical travel distance, and thus the distribution of the average travel time, which results in the vertical mixing of the electrons.

By assuming that the distribution of the penetration depth is exponential and that of the travel time is asymptotically approximated by a Gaussian distribution, the variance of the arrival time of the signal electrons was derived as follows [1]:

*σ _s_*^2^ = *σ _m_*^2^ + *σ _d_*^2^, (1)

where

*σ _m_*^2^ = −*δ*^2^*W* ’^2^/(*p*^2^*v*^2^) exp (−*W* ’) + *δ*^2^/*v*^2^, (2)

*σ _d_*^2^ = {(*W* ’ − *p*)/*p*} (2D*δ*/*v*^3^), (3)

where

*P* = 1 − exp (−*W* ’), *W* ’ = *W/δ*, (4)

where *σ* *_m_* ^2^, *σ* *_d_* ^2^ and *σ* *_s_* ^2^ are variances due to the mixing effect, the diffusion effect, and the total effect, respectively, *W* is the width of the chip, *δ* is the penetration depth, *v* is the drift velocity, and *D* is the vertical diffusion coefficient. When the values of the four parameters, *v*, *D*, *δ* and *W*, are assigned, the value of the standard deviation, *σ* *_s_* ^2^, is calculated.

The conditions to minimize Equation (1) were thoroughly discussed. For example, the drift velocity appears in the denominators in Equation (1) with the power of 2 and 3. Therefore, the increase of the drift velocity effectively decreases the temporal resolution limit. The drift velocity is increased by a higher field. However, as is known, the value is saturated and approaches a constant value at a critical value of the field. On the other hand, the diffusion coefficient decreases for the higher field and takes the minimum at the critical field value, which is 25 kV/cm for the crystal orientation of the intrinsic silicon <111> at 300 K. The combination of the intrinsic silicon and the <111> crystal orientation provides the combination of the values of the drift velocity and the diffusion coefficient to minimize Equation (1).

For *W* ’ = *W/δ* ≪ 1, most of incident photons penetrate through the chip, resulting in a very low sensitivity. The loss of sensitivity is crucial in the ultra-high-speed imaging. If *W* ’ ≫ 1, the temporal resolution limit becomes low, as seen from Equation (1). Therefore, the condition of *W* ’ = 1 may be reasonably accepted.

For *W* ’ = 1 with the wavelength of the incident greet light (λ) of 550 nm and the temperature of 300 K, Equation (1) provides a temporal resolution limit of Δt equal to 12.42 ps. The value was calculated by using the values of *v* and *D* estimated by the Monte Carlo simulation. When the values of the experimental results are substituted into Equation (1), ∆t was 11.1 ps (equivalent to the frame rate of 90.1 Gfps).

### 3.2. Trade-Offs from Theory to Practice

The frame rate closer to the theoretical limit may be achieved by designing the sensor based on the conditions introduced to minimize the spread of the distribution of the arrival time, including:

(1) suppressing the effect of the horizontal motion of the signal electrons,
(2) application of the critical field,
(3) the ratio of the width to the penetration depth of the chip (*W* ’) equal to 1,
(4) use of a wafer with the silicon crystal orientation <111>, and
(5) incident light perpendicular to the backside.

However, some of these conditions conflict with important performance indices, such as sensitivity and crosstalk, other than the frame rate. Therefore, the trade-off between each of these conditions for the highest theoretical frame rate and the corresponding conflicting performance indices should be adjusted in practical design of the sensor. Table 3 lists the conditions together with the countermeasures to mitigate the trade-offs. It should be noted that the <111> wafer can slightly improve the temporal resolution compared with the common <100> wafer.

For example, a simple way to suppress the effects of the horizontal motion is focusing the incident light in the center of the backside of each pixel with a microlens and/or a light guide and guiding the generated electrons to the front side through a narrow cylindrical pipe.

The deep trench insulator (DTI) may be a good candidate to make the narrow pipe. Advanced BSI image sensors are equipped with the DTI, along the pixel boundary to suppress spatial crosstalk among the pixels. However, for the proposed application, the DTI is installed at the far inside of the pixel. A new technology always involves new difficulties. For the narrow DTI pipe, there are two disadvantages:

(1) large dark current: unlike the DTI used in the standard BSI image sensors, the inside of the narrow DTI pipe must be depleted to apply the vertical critical field to improve the temporal resolution. The depletion causes a large dark current from the side wall of the DTI;
(2) loss of incident light: it is difficult to perfectly focus the incident light in the narrow pipe area, especially at pixels near the edge of the photo-receptive area with tilted incident light.

The large dark current may not be serious for the ultra-high-speed imaging. However, it should be carefully pre-evaluated when in-pixel signal accumulation is introduced for a very small number of photons per a pixel per a frame, which is typical in ultra-high-speed imaging of reproducible events.

The condition of *W* ’ = *W*/*δ* = 1 is another example of the trade-off. The crosstalk due to the MCG structure is unavoidable, although the effect can be compensated by post-digital data processes [9,10]. When *W* ’ = 1, a large proportion of incident photons (36.8%) reach the front side, enhancing the crosstalk.

The condition of *W* ’ = 3, is a simple method to mitigate the problem with the remaining photons at the front side less than 5%. For example, for *W* ’ = 4.05 (*W* = 7 µm, *δ* = 1.73 µm) with λ = 550 nm and *W* ’ = 1.75 (*W* = 7 µm, *δ* = 4.00 µm) with λ = 650 nm, the average of *W* ’ for these two cases is 2.90. Hence, through the green to the red light, the condition of *W* ’ = 3 is approximately satisfied, sacrificing the temporal resolution.

**Figure 9:**
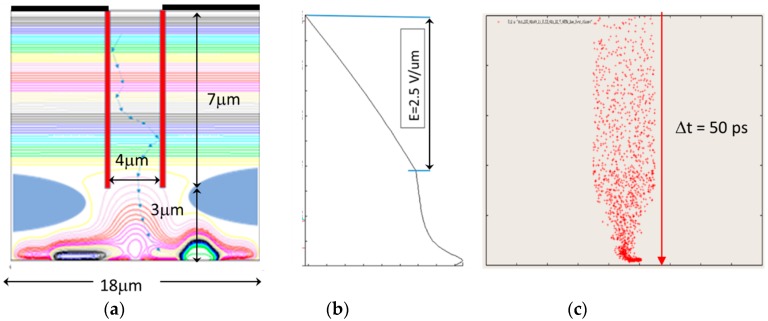
Simulation results of the design method of an ultra-high-speed image sensor based on the theoretical analysis of the temporal resolution limit.

Incident light with the distributed angle even shortens the average penetration depth. This effect can be easily introduced by modifying the value of the penetration depth. Therefore, these conditions were excluded from the following preliminary evaluation. A simple example of the sensor, shown in Figure 9a,b, was prepared for evaluating the effectiveness of the DTI pipe. Figure 9c shows the simulation result.

The simulation assumed that the generated electrons are reflected by the wall of the DTI, although they are actually repulsed by the weak negative potential built with Boron ions near the wall.

The result shows that the horizontal motion is effectively suppressed, and an improved temporal resolution of 50 ps is achieved.

## 4. Conclusions

The paper summarized the current status of the research and development of the BSI MCG image sensor. The temporal resolution of 10 ns has been achieved, and can reach 50 ps with proper design of the sensor with theoretical considerations on the temporal resolution limit.

The sensor can be applied not only to ultra-high-speed imaging, such as imaging of fast cruck propagation and blasting, but also to innovation of advanced analyses and measurement technologies requiring ultra-high-speed image sensors, such as FLIM, imaging TOF-MS, and LIDAR.

## Figures and Tables

**Table 1:** Fundamental performance of silicon image sensors (expressed by round numbers).

Resolution				Dynamic Range
	A Ultimate	B State-of-the-Art	B/A	
Sensitivity	1 photon (1 e^−^) *	1 e^−^ (0.3 e^−^ rms) *	1	SN ratio
Spatial	0.3 µm **	1 µm	3	Pixel Count
Temporal	10 ps	10 ns ***	1000	Frame Count

* If the quantum efficiency is one, one photon generates one electron. To detect one electron at a sufficiently high confidence level, the readout noise level of about 0.3 e^−^ rms is required [8]. ** The Rayleigh criterion for the wavelength of 550 nm. *** The achievement presented in this paper. Photon-counting-type image sensors, such as SPAD, have achieved 100 ps [9]. However, repetitive imaging is necessary to get an image with these sensors [4].

**Table 2:** Specification of the test sensor.

Structure	BSI MCG (Backside-Illuminated Multi-Collection-Gate) Image Sensor
Shortest frame interval (equivalent frame rate)	10 ns (100 Mfps)
Frame and pixel counts	5 frames for 576 × 512 × 2 pixels * 10 frames for 576 × 512 pixels
Fill factor	100%
Pixel size	12.73 µm × 12.73 µm *
Photoreceptive area	10.368 mm × 9.216 mm *
Process	130 nm CMOS process modified for CCD

* The pixels are staggered. The pitch of the even-column pixels is 18 µm, and the edge length is 12.73 µm (see Figure 1).

**Table 3:** Modification of conditions used in the theoretical analysis for designing practical sensors.

Reasons of Introduction	Terms in the Theoretical Analysis		Practical Countermeasures	
	Elements	Employed Conditions	Measures to Mitigate	Trade-Offs/Side Effects
To enhance the temporal resolution	Horizontal motion	Reduction	(Microlens/light guide) + (DTI/p-well)	Less/non-uniform sensitivity, Dark current
	Thickness of the layer (*W*)	= Penetration depth (*δ*)	*W* ’ = *W*/*δ* = 3	Crosstalk vs. Temporal resolution
	Electric field	25 kV/cm	-	Dark current
	Incident light direction	Perpendicular to the backside	Adjustment for the distributed light direction	-
	Crystal orientation	<111>	<100>	-
	Impurity concentration	0 (intrinsic)	10^14^ cm^−3^	-
For formulation	Arrival-time distribution	Gaussian distribution	-	-
	Definition of the resolution	Ditto:|*µ*_2_ − *µ*_1_| = 2 *σ*	-	-
